# Investigation of a Simple Model for Within-Flock Transmission of Scrapie

**DOI:** 10.1371/journal.pone.0139436

**Published:** 2015-10-01

**Authors:** Thomas J. Hagenaars, Jack J. Windig

**Affiliations:** 1 Central Veterinary Institute, part of Wageningen UR, P.O. Box 65, 8200 AB Lelystad, The Netherlands; 2 Wageningen UR Livestock Research, Animal Breeding and Genomics Centre, P.O. Box 338, 6700 AH Wageningen, The Netherlands; The University of Tokyo, JAPAN

## Abstract

Genetic control programs for scrapie in sheep build on solid knowledge of how susceptibility to scrapie is modulated by the prion protein genotype at the level of an individual sheep. In order to satisfactorily analyze the effectivity of control programs at the population level, insight is needed at the flock level, i.e., how the grouping of sheep in flocks affects the population-level transmission risk. In particular, one would like to understand how this risk is affected by between-flock differences in genotype frequency distribution. A first step is to model the scrapie transmission risk within a flock as a function of the flock genotype profile. Here we do so by estimating parameters for a model of within-flock transmission using genotyping data on Dutch flocks affected by scrapie. We show that the data are consistent with a relatively simple transmission model assuming horizontal transmission and homogeneous mixing between animals. The model expresses the basic reproduction number for within-flock scrapie as a weighted average of genotype-specific susceptibilities, multiplied by a single overall transmission parameter. The value of the overall transmission parameter may vary between flocks to account for random between-flock variation in non-genetic determinants such as management practice. Here we provide an estimate of its mean value and variation for Dutch flocks.

## Introduction

Classical scrapie in sheep is a disease that potentially can be controlled by selective breeding, due to the high to full scrapie resistance of certain genotypes. Scrapie is a transmissible spongiform encephalopathy (TSE) with an incubation period of one or more years before the occurrence of clinical signs, such as uncoordinated movement, abnormal postures and severe scratching. The susceptibility to scrapie is modulated by polymorphisms of the sheep prion protein (PrP) gene [1–5]. The most important polymorphisms occur at the codons 136, 154 and 171. Five alleles (VRQ, ARQ, AHQ, ARH and ARR) are observed in The Netherlands. The VRQ allele is known to confer high susceptibility to classical scrapie, the ARQ and ARH alleles are associated with moderate susceptibility and the AHQ allele with low susceptibility. The ARR allele confers resistance, with the homozygous genotype ARR/ARR being extremely resistant. These properties make the use of exclusively ARR/ARR rams for breeding a means to breed selectively for scrapie resistance. European Union (EU) regulation since 2001 requires the selection of rams intended for breeding in scrapie-free flocks of “high genetic merit” (followed by culling of the rams with a VRQ allele). A further important scrapie control activity is the surveillance programme of testing healthy slaughtered sheep and fallen stock for scrapie by a rapid test on brainstem samples. This programme concerns animals over 18 months of age and was introduced in the EU in 2002 [6]. Since 2003, the EU requires control measures in flocks of origin of classical-scrapie positive animals in the active or passive surveillance. These measures consist of either a whole-flock cull or genotyping all animals and culling the animals of susceptible genotype and examining the brain stem of all or a sample of the culled animals of at least 12 months of age for scrapie positivity, using rapid tests.

Some member states have introduced a wider national breeding programme than requested by the EU, including The Netherlands (started in 1998), Great Britain (started in 2001) [7–11], and France (started in 2002). As these programs have to be run over many years there is a need for reliable model projections of their expected future effects [12]. In such predictive model analyses it is often desirable to quantify within-flock scrapie transmission as a function of flock genotype profile. No such quantification has been performed to date, although a statistical analysis of 30 affected flocks by McKintyre et al. [13] provided evidence that the flock genotype profile is correlated with outbreak characteristics such as mean yearly incidence, and Tongue at al. [14] identified PrP genotype and allele frequencies as flock-level risk factors for scrapie in a case-control study comprising 293 flocks. Here we use data that has accumulated due to EU statutory measures, that include the genotyping and (partial) culling and testing of flocks of origin of scrapie-positive animals. Using the Dutch culled-flocks data, we show here how this type of data can be used to calculate the basic reproduction number for within-flock transmission. We find that the data are consistent with a relatively simple transmission model assuming horizontal transmission and homogeneous mixing between animals. The model expresses the basic reproduction number for within-flock transmission of scrapie as a weighted average of genotype-specific susceptibilities, multiplied by a single parameter that is drawn from a distribution to account for random between-flock variation in non-genetic determinants such as management practice.

## Materials and Methods

### Culled-flocks data

The Dutch culled-flocks data (2003–2008) consist of scrapie genotyping results and scrapie infection test results in animals that were culled, as part of the mandatory scrapie control efforts, on 69 flocks of origin of scrapie index cases. The data is included as S1 Dataset. Immunohistochemistry (IHC) was used for confirmation of the positive cases detected using the rapid test. IHC and Western blotting were used to discriminate between classical and atypical scrapie. PrP genotypes were determined (at codons 136, 154, and 171) by a routine TaqMan test that is completely automated. It can detect polymorphisms 136 A to V, 154 R to H, and 171 Q to R. From 2006 onwards our TaqMan genotyping additionally distinguishes between Q and H at codon 171. When analyzing the culled-flocks data we consider total numbers of animals for each genotype across the period 2003–2008 and we therefore group the 2006–2008 ARQ and ARH results together, using the notation ARQ*. The TaqMan principle is a test in which a small part of the PrP gene is amplified. During amplification dedicated fluorescent probes are used to detect absence/presence of specific polymorphisms. A second test, based on pyro-sequencing, was used as a confirmatory test on randomly selected samples. The rapid tests used were the Prionics Check Western (2002–2006) and the Prionics Check Western SR from June 2006 onwards. Data statistics such as the overall genotype and allele frequencies across the 69 culled flocks as well as the mean detected scrapie prevalence by genotype are given in Ref. [15].

### Transmission model

Our model relating within-flock basic reproduction number to the genotype distribution is a simplified version of the more general model structure described by Hagenaars et al. [16]. The most important simplification is to refrain from a stratification by age, a necessary simplification because we do not have age information on the animals tested in the culled-flocks data. The model takes the form of a genotype specific SI (susceptible-infected) model with *S*_*γ*_ and *I*_*γ*_ being respectively the proportion of animals in the flock that are susceptible and infected and have genotype *γ*. The change of *S*_*γ*_ and *I*_*γ*_ are modelled as follows:

$$\begin{array}{l} \frac{dS_{\gamma }}{dt}=\mu f_{\gamma }-\beta g_{\gamma }S_{\gamma }\sum_{\gamma '}h_{\gamma '}I_{\gamma '}-\mu S_{\gamma }\\ \frac{dI_{\gamma }}{dt}=\beta g_{\gamma }S_{\gamma }\sum_{\gamma '}h_{\gamma '}I_{\gamma '}-\mu I_{\gamma } \end{array}$$

Here the change in *S*_*γ*_ is due to the new(born) animals coming into the flock minus suspected animals becoming infected and minus suspected animals being replaced. The change in *I*_*γ*_ is due to suspected animals becoming infected and infected animals being replaced. The term *μf*_*γ*_ describes the recruitment of new(born) animals of genotype *γ* into the flock, with *μ* being the replacement rate of animals and *f*_*γ*_ = *S*_*γ*_ + *I*_*γ*_ being the frequency (proportion) of animals in the flock that has genotype *γ*, a frequency which is assumed to be (quasi-)stationary. This description applies to situations where no animals are bought in (closed flock), or where bought-in animals are recruited from a population with the same genotype frequency distribution. The parameter *β* is a transmission rate parameter; the term proportional to *β* represents the rate of infection transmission to animals of genotype *γ*, *g*_*γ*_ the relative susceptibility of genotype *γ*, and *h*_*γ*_ an infectiousness parameter. The relative susceptibility *g*_*γ*_ is defined as the susceptibility relative to that of the reference genotype *γ*_*R*_ = ARQ*/VRQ (i.e. setting $g_{\text{ARQ}*/\text{VRQ}}=g_{\gamma_{R}}=1$). The infectiousness parameter *h*_*γ*_ is introduced in order to account for between-genotype differences in how infectious an infected animal is to its flock mates. The terms *μS*_*γ*_ and *μI*_*γ*_ are the rates of replacement of animals. As susceptible and infected individuals are subject to the same replacement rate *μ*, the model neglects any scrapie-related mortality (or preferential replacement). The analysis of Matthews et al. [17] shows that vertical scrapie transmission is estimated to make only a minor contribution to the total transmission. We therefore neglect the vertical route here, as its incorporation would make the modelling considerably more complex. In this model the within-flock basic reproduction number for scrapie transmission, denoted here as $R_{0}^{\text{w}}$, where the superscript ‘w’ is referring to “within-flock”, is expressed in terms of the genotype distribution *f*_*γ*_ as follows:

$$R_{0}^{\text{w}}=\rho_{0}\sum_{\gamma }f_{\gamma }g_{\gamma }h_{\gamma }.$$

Here the basic reproduction number is defined as the expected number of new infections in the flock caused by a single typical primary scrapie infection in the limit of negligible infection prevalence; this corresponds to the standard textbook definition [18]. The parameter *ρ*_0_ is defined as ρ0=βμ; it serves as a transmission scale parameter and can be interpreted as a base-line value of the reproduction number corresponding to a hypothetical situation in which the flock comprises a single genotype with relative susceptibility 1. The above expression for $R_{0}^{w}$ can be obtained by employing a next-generation operator approach, and by using the observation that the operator has one-dimensional range (or equivalently: mixing is separable) as explained in Ref. [18], section 7.4.1. We set the infectiousness parameter *h*_*γ*_ equal to one for all genotypes without ARR allele, thus assuming that these genotypes, if infected, have the same infectiousness. For those with at least one ARR allele it is set to zero, i.e. in particular we assume that the contribution of ARR/VRQ animals to *R*_0_^*w*^ is negligible. This assumption is motivated by the observation that the pathogenesis in this genotype does not (or only minimally) affect the lymphoreticular system [19]. (ARR/VRQ sheep are present in 59 out of 69 culled flocks. Overall the ARR/VRQ frequency in the culled flocks was 7.1 percent (Table 6 of Ref. [15])).

In order to incorporate variation in $R_{0}^{w}$ due to causes different from the genetic content of the flock, the parameter *ρ*_0_ is taken to be a distributed quantity (i.e. randomly varying between flocks). Such causes may include farm type and management practices in particular during the lambing period (e.g. using lambing pens or not). Values for the relative susceptibilities *g*_*γ*_ are obtained from the estimates of the detected infection prevalence in the different genotypes across all culled flocks by Hagenaars et al. [15] and listed in Table 1. We calculate the relative susceptibility from this genotype-specific prevalence, denoted by $\bar{I_{\gamma }}$, using the following relationship that is derived in S1 Text:

$$g_{\gamma }=\frac{\bar{I_{\gamma }}}{\bar{I_{\gamma_{\text{R}}}}}\left(\frac{1-\bar{I_{\gamma_{\text{R}}}}}{1-\bar{I_{\gamma }}}\right).$$ (1)

Here $\bar{I_{\gamma_{\text{R}}}}$ denotes the prevalence in the reference genotype. We use the genotype ARQ*/VRQ, being the most frequent genotype amongst Dutch scrapie cases, as this reference. Only the genotypes without ARR allele are relevant, as the others do not contribute to $R_{0}^{w}$ due to *h*_*γ*_ being zero. As noted in Ref. [15], infection prevalence is approximately proportional to susceptibility when prevalence is low. The relationship (1) takes into account the non-linearity of the relationship for intermediate and high prevalence, based on approximating the dataset as one single flock. In our model we for simplicity replace the estimate *g*_AHQ/ARQ*_ = 0.013 by *g*_AHQ/ARQ*_ = 0. Due to the low frequency of the AHQ/ARQ* genotype and its low estimated relative susceptibility this is a good approximation.

**Table 1:** Genotype-specific scrapie risk and corresponding estimates for the relative susceptibility. Estimates, obtained in Ref. [15] from culled-flocks data, of the genotype-specific risk of being tested scrapie positive, for genotypes (or groups of genotypes) without ARR allele, relative to the ARQ*/VRQ group of genotypes.

Genotype	Relative scrapie risk (Confidence bounds)	Relative susceptibility estimate
AHQ/ARQ*	0.02 (0.001 ‒ 0.08)	0.013
AHQ/VRQ	0.27 (0.05 ‒ 0.79)	0.21
ARQ*/ARQ*	0.10 (0.08 ‒ 0.14)	0.08
ARQ*/VRQ	1.00 (1.00 ‒ 1.00)	1
VRQ/VRQ	1.31 (0.88 ‒ 1.83)	1.50

Due to the absence of age structure, our model is simpler than previously published within-flock scrapie transmission models. Those publications were typically dealing with individual outbreaks for which more detailed data was available, and were reviewed in Ref. [12]. The mathematical structure of the models of most previous work (including Refs. [16,17,20–22]) reduces to that of our model when the age structure is left out, clinical onset is left implicit and disease-induced mortality is neglected.

### Testing the model

The model described above assumes that, whereas the value of the transmission scale parameter *ρ*_0_ varies between flocks, the relative susceptibility parameter *g*_*γ*_ is the same for all flocks. We seek to validate this assumption against data of culled flocks., The assumption implies that the genotype-dependent infection risk *p*_*γ*,*i*_ in flock *i* can be expressed in terms of a flock-dependent base-line (ARQ*/VRQ) risk *p*_ARQ*/VRQ,*i*_ and a flock-independent parameter *g*_*γ*_ as follows:

$$p_{\gamma ,i}=\frac{g_{\gamma }p_{\text{ARQ}*/\text{VRQ},i}}{\left(1-p_{\text{ARQ}*/\text{VRQ},i}\right)+g_{\gamma }p_{\text{ARQ}*/\text{VRQ},i}}.$$ (2)

Eq (2) is derived in S1 Text. The actual within-flock incidence by genotype would arise from a binomial distribution with probability *p*_*γ*,*j*_.

To test whether the model is adequate we investigated for each culled flock *i* with secondary cases and with at least one animal of ARQ*/VRQ genotype tested (46 flocks in total), whether it is possible to choose a value for *p*_ARQ*/VRQ,*i*_ such that for all genotypes without ARR allele the observed data are within the 95% probability range of the binomial model using *g*_*γ*_ estimated at population level (and listed in Table 1).

### Parameter estimation

For the estimation of parameters we assume that the transmission dynamics in the flocks was in an endemic phase, i.e. in (quasi-)stationary equilibrium. This assumption is necessary due to the absence of longitudinal information on prevalence. It represents an approximation, as within-flock dynamics is expected to be out of (quasi-)equilibrium for at least the early and late parts of the outbreak period. We believe that for culled flocks with at least one detected secondary scrapie case, this approximation is justifiable due to the dominance of the quasi-stationary part of outbreaks indicated by the modelling results in figure 2 of Ref. [21]. In endemic equilibrium the model equations provide a relationship between the parameter *ρ*_0_ and the proportion infected i* of animals without ARR allele as follows:

$$i^{*}-\sum_{\gamma \in \Gamma }f_{\gamma }\left(1-\frac{1}{\left(1+\rho_{0}g_{\gamma }i^{*}\right)}\right)=0$$ (3)

Here Γ is the set of genotypes without ARR allele; this relationship is derived in S1 Text. For a subset of culled flocks, we use the above equation to estimate *ρ*_0_ for each flock separately by assuming the flock is in endemic equilibrium. In order to use these results for modelling the between-flock variation in the parameter *ρ*_0_ due to flock-specific aspects different from the genetic content of the flock, we fit a Weibull distribution to the histogram of estimates. We relate the proportion i* to the proportion *i* ^+^ found positive of tested animals without ARR allele by assuming that $i^{*}=\frac{i^{+}}{Se}$, with *Se* the (unknown) test sensitivity in detecting scrapie infection in animals without ARR allele of at least 12 months of age in endemically affected flocks. Below we motivate our approximation of assuming *Se* to be independent of genotype. We consider different values of *Se* across the range [0.55–0.95] to analyze the influence of this parameter on our results, choosing *Se* = 0.75 as a default value. This range is motivated by the sensitivity of the test of close to 95% as evaluated on scrapie cases confirmed by Western Blot of the brainstem [23] and the notion that early on in the incubation period scrapie infection has not yet propagated to the brainstem [24]; detected scrapie prevalence in the culled flocks suggests that a sensitivity below 0.55 is unlikely. The subset of flocks is obtained by requiring that at least two positive cases were found and in addition that at least eight animals of the genotype ARQ*/VRQ (the most common genotype amongst Dutch cases [15]) in this flock were tested. The first requirement was made because with only one detected case the assumption of endemicity was deemed too crude, and the second requirement was made in order to avoid that the estimated endemic prevalence in the flock (Eq (3)) became dominated by noise.

We use a genotype-independent parameter *Se* for the sensitivity of the rapid test. In general the sensitivity of the scrapie test may be expected to depend on genotype, as the sensitivity depends on how far the animal has progressed towards clinical onset [19,24–27], and the incubation period is dependent on the genotype. However, as can be seen from the age-at-onset results in the Electronic Supplementary Material of the paper by Gubbins [25], in fact the incubation period distributions for the three genotypes without ARR allele (ARQ/ARQ, ARQ/VRQ, en VRQ/VRQ), are very similar. This motivates our choice to use the approximation of a genotype-independent test sensitivity. In case new data would provide evidence for a genotype dependent sensitivity of the tests used, this could of course be included in a revised analysis. We note that in the context of a rectal biopsy test evidence for such a dependence has been found [28].

### FIS values and back-calculating genotype distributions

During the period 2003–2008 in which the flock cull data was gathered, selection for ARR alleles was ongoing in The Netherlands: E.g., from October 2004 onwards the use of ARR/ARR rams was obligatory for flocks with more than 10 breeding ewes (except some rare breeds), and from September 2005 until June 2007 the use of ARR/ARR rams was obligatory for all flocks (except some rare breeds) [15,29]. This forms a complication for our analysis because, if selective breeding takes place on an infected flock, genotype and allele frequencies found at the time of flock culling are not representative of the frequencies at the time that the scrapie infection became established in the flock. This latter “original” profile is the relevant one for our analysis of the basic reproduction number. To detect a possible history of selection for ARR alleles, we compare observed to expected heterozygosities for each flock. In the absence of selection genotype frequencies are expected to be in Hardy-Weinberg equilibrium. I.e. if the resistant allele R occurs in the population with frequency p and the susceptible allele S with frequency 1-p, the frequency of the homozygous genotype R/R is expected to be p^2^, the frequency of S/S to be (1-p)^2^ and the frequency of the heterozygous genotype R/S to be 2p(1-p). The deviation of Hardy-Weinberg equilibrium within a flock can be quantified by calculating the FIS value [30]: FIS = (*H*_obs_ − *H*_exp_)/*H*_exp_, with *H*_obs_ being the observed frequency of heterozygotes in the flock and *H*_exp_ the expected frequency of heterozygotes based on the allele frequencies in the flock assuming Hardy-Weinberg equilibrium. Negative FIS values (quantifying overrepresentation of heterozygote genotypes in comparison to expected frequencies when assuming Hardy-Weinberg equilibrium) indicate recent selective breeding.

In case of recent selective breeding we need to back-calculate from the current genotype profile the profile at the time that the current scrapie cases became infected. To this end, we assume that the original distribution is in Hardy-Weinberg equilibrium, and that the selective breeding was carried out by using ARR/ARR rams. In that case, as shown in S1 Text, we can derive the following analytical relationship between the original equilibrium value ${\bar{f}}_{ARR}$ of the ARR allele frequency and the frequencies $f_{R/R}^{c}$ of resistant animals and $f_{S/S}^{c}$ of non-ARR carrying animals at the moment of culling:

$${\bar{f}}_{ARR}=\frac{1}{2}\left(1-f_{S/S}^{c}+f_{R/R}^{c}-\sqrt{{\left(1-f_{S/S}^{c}+f_{R/R}^{c}\right)}^{2}-4f_{R/R}^{c}}\right).$$ (4)

For flocks with negative FIS value we use Eq (4) to back-calculate. For consistency we also use equilibrium values for the genotype frequencies of flocks with a positive FIS value; in this case by calculating the Hardy-Weinberg equilibrium genotype frequencies corresponding to the allele frequencies at the moment of culling. We note that whereas these back-calculations do change the frequency of the group of non-ARR carrying (S/S) animals, the relative proportions of the different genotypes within this group remain unchanged. This implies that when testing the model assumption of flock-independent relative risks in non-ARR genotypes (as described above) we can work with the culled-flocks data before back-calculation.

## Results

### One example flock

To illustrate the analyses, we consider as an example a positive flock detected and culled in 2007. For this flock, 417 animals were genotyped and out of these, 200 were tested. Out of 21 ARQ*/VRQ, 55 ARQ*/ARQ*, 3 AHQ/VRQ, 19 AHQ/ARQ*, and 14 ARR/VRQ animals tested, 11 in total were found scrapie positive (including index case): 9 ARQ*/VRQ, 1 ARQ*/ARQ* and 1 AHQ/ARQ*. When we set *p*_ARQ*/VRQ_, the scrapie risk for ARQ*/VRQ, equal to 9/21≈43% and multiply this with the relative risks of Table 1 to obtain expected case frequencies of the other genotypes, we find that for all these genotypes the actual case numbers are within the 95% probability range of a binomial model. For this flock the FIS value was negative (-0.08), possibly due to recent selective breeding. Based on the Hardy-Weinberg equilibrium genotype frequencies calculated using Eq (4), and setting *Se* to its best-fit value (see below) we estimated *ρ*_0_ = 8.3 and $R_{0}^{\text{w}}$ = 1.3 for this flock. We now describe the overall results.

### Testing the model

We find that for 42 out of the 46 flocks with secondary cases and with at least one animal of ARQ*/VRQ genotype tested, it is possible to choose a value for *p*_ARQ*/VRQ,*i*_ such that for all genotypes the observed data are within the 95% probability range of the model using the relative susceptibility *g*_*γ*_ estimated at population level. This analysis yielded values for *p*_ARQ*/VRQ_ ranging from 4% to 100% with a median of 43%. Amongst the four flocks outside the 95% probability range of the model, two flocks are outside the range due to having ARQ*/ARQ* positives but no ARQ*/VRQ positives, suggestive of an “ARQ” adapted scrapie strain with different relative susceptibilities. No evidence of a more widespread presence of an ARQ adapted strain was found: In only two of the 42 flocks consistent with the relative susceptibility model the detected prevalence in ARQ*/ARQ* exceeded that in ARQ*/VRQ; both had low numbers of tested ARQ*/VRQ animals. From this we conclude that the data is broadly consistent with our binomial model assuming flock-independent relative risk.

FIS values

**Fig 1:**
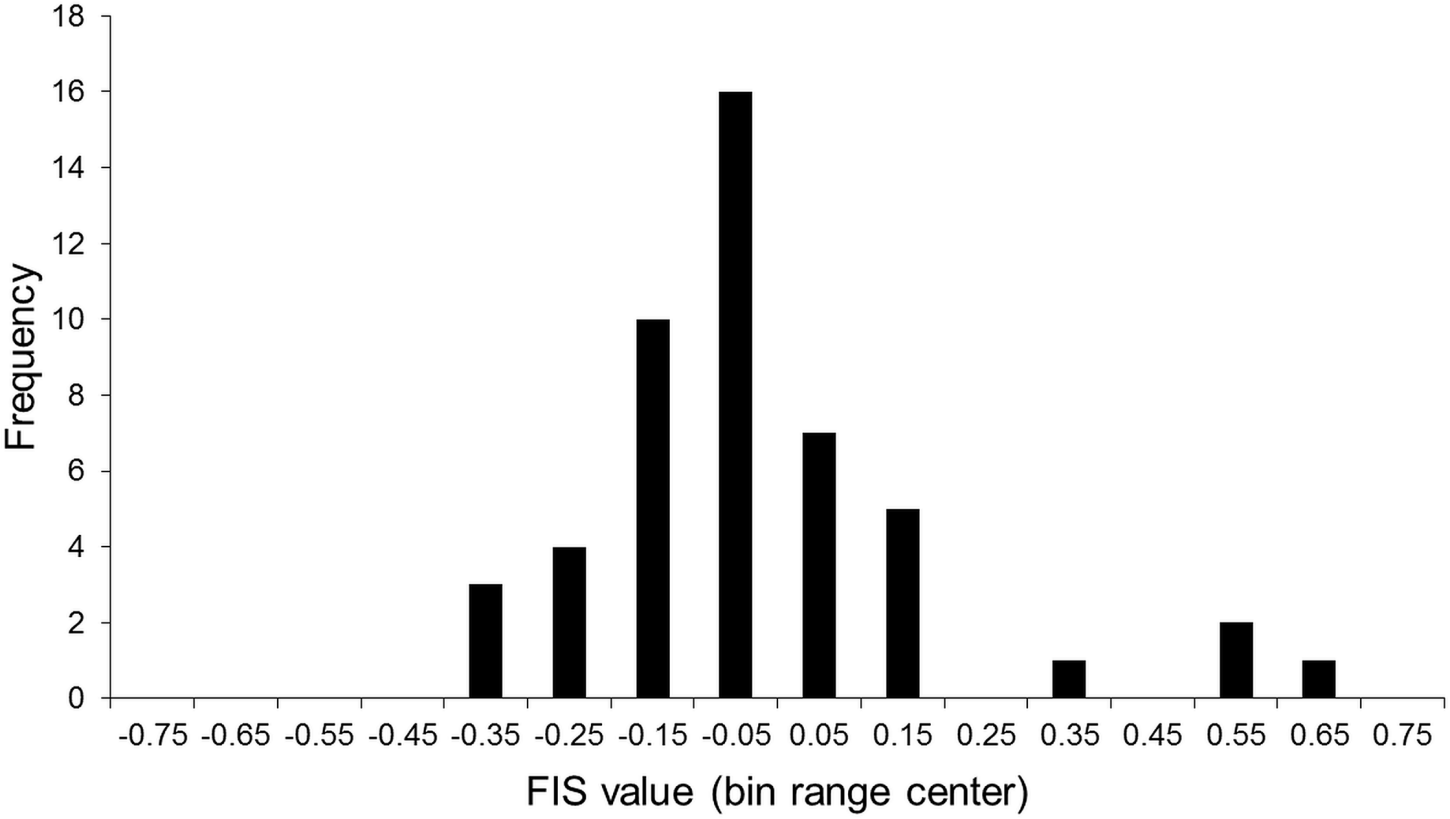
FIS values histogram. Histogram of the FIS values calculated for 49 culled flocks with at least one secondary scrapie case. Negative FIS values, representing a heterozygote frequency excess, occur for 33 flocks.

In Fig 1 we show a histogram of the FIS values calculated for the genotype frequencies at the moment of culling for 49 flocks with at least one secondary scrapie case. Negative FIS values, representing a heterozygote frequency excess, occur for 33 flocks, indicating a recent history of selective breeding in these flocks.

### Parameter estimation

**Fig 2:**
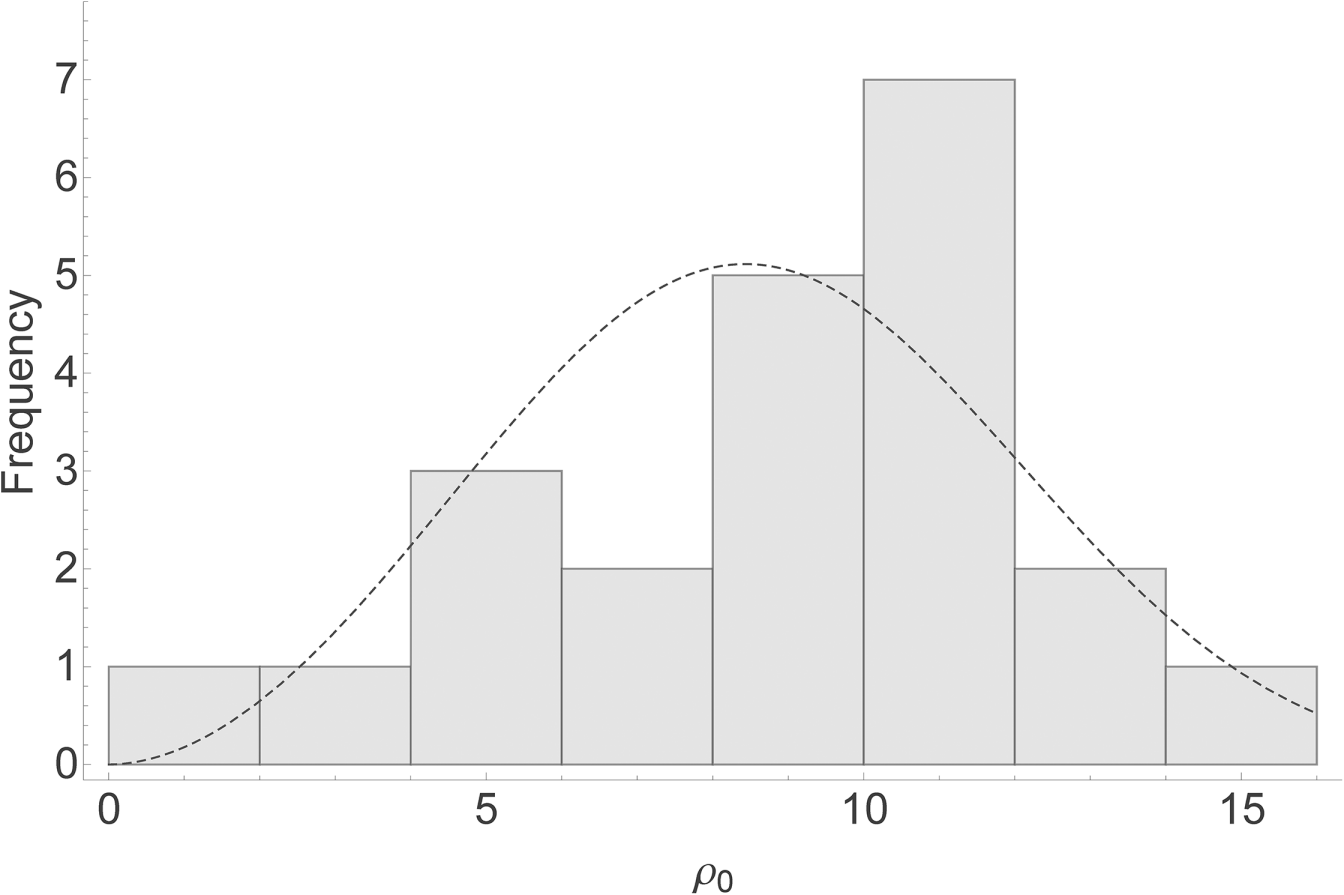
Scale parameter distribution. Histogram of 22 estimated values for the transmission scale parameter *ρ*_0_. The dashed line is a Weibull frequency distribution with mean and variance equal to the mean and variance of the *ρ*_0_ estimates for *Se* = 0.75.

**Fig 3:**
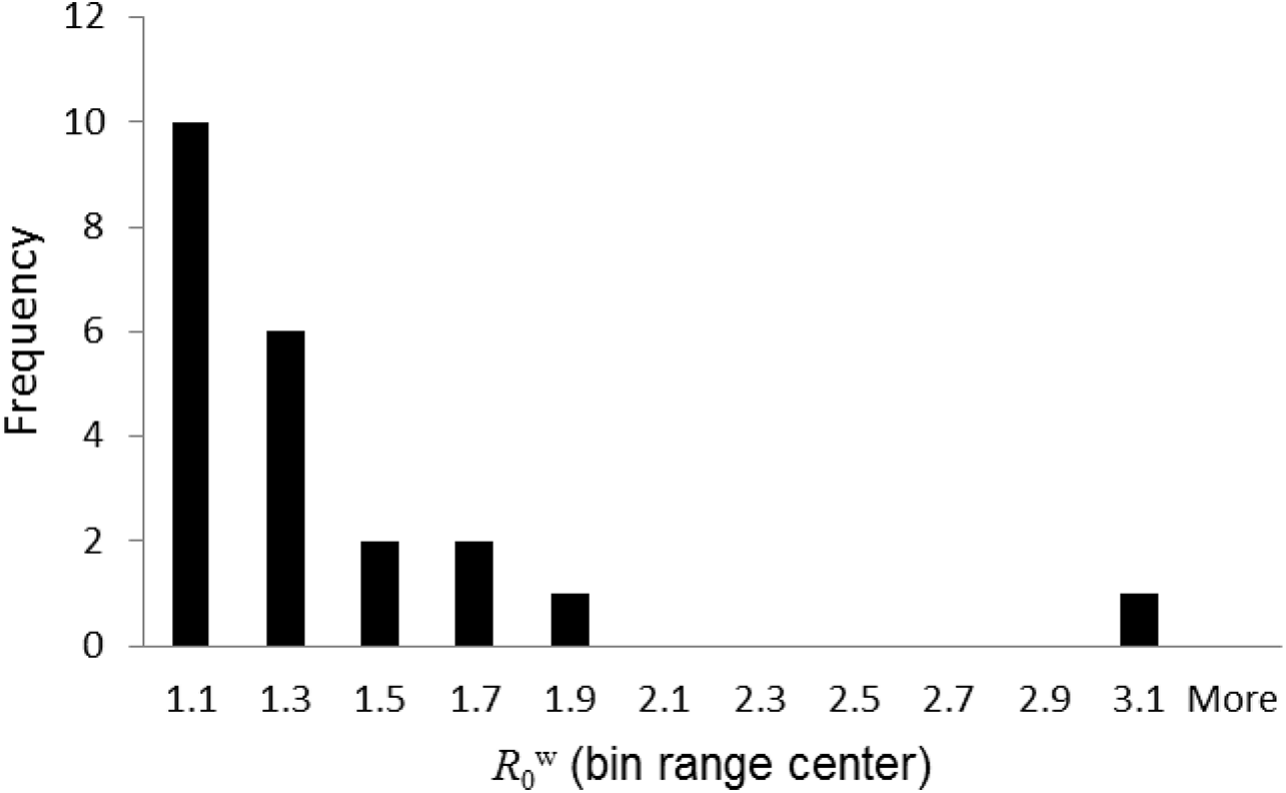
Distribution of the within-flock reproduction number. Histogram of 22 estimated values for the reproduction number $R_{0}^{w}$.

**Fig 4:**
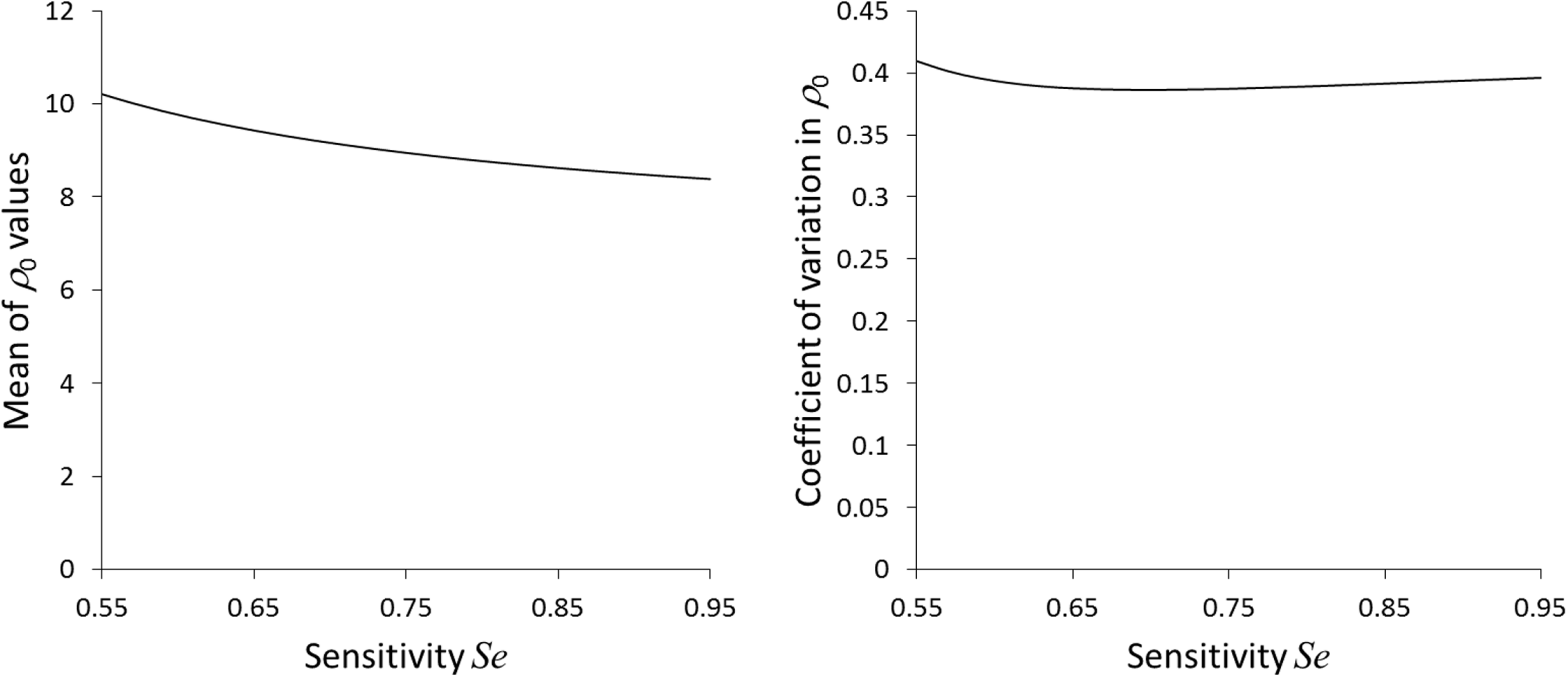
Dependence on sensitivity parameter. Mean (left-hand panel) and coefficient of variation (right-hand panel) of estimated *ρ*_0_ values as a function of the sensitivity *Se*.

The subset of flocks with at least two positive cases and at least eight tested animals of genotype ARQ*/VRQ contained 22 flocks, out of which 17 had a negative FIS value. In Fig 2 we plot a histogram of the estimated values for the transmission scale parameter *ρ*_0_ for these 22 flocks, based on a value for the sensitivity parameter of *Se* = 0.75. The corresponding histogram of $R_{0}^{w}$ values is presented as Fig 3. In Fig 2 we also plot a Weibull frequency distribution with mean and variance equal to the mean and variance of the *ρ*_0_ estimates. In Fig 4 we show how mean and variance of the distribution of *ρ*_0_ values change with the value assumed for *Se*. We observe that both the mean $\bar{\rho_{0}}$ and the variance of the estimated *ρ*_0_ values are only weakly sensitive to the assumed *Se*, in other words that our results are robust against the uncertainty in *Se*.

**Fig 5:**
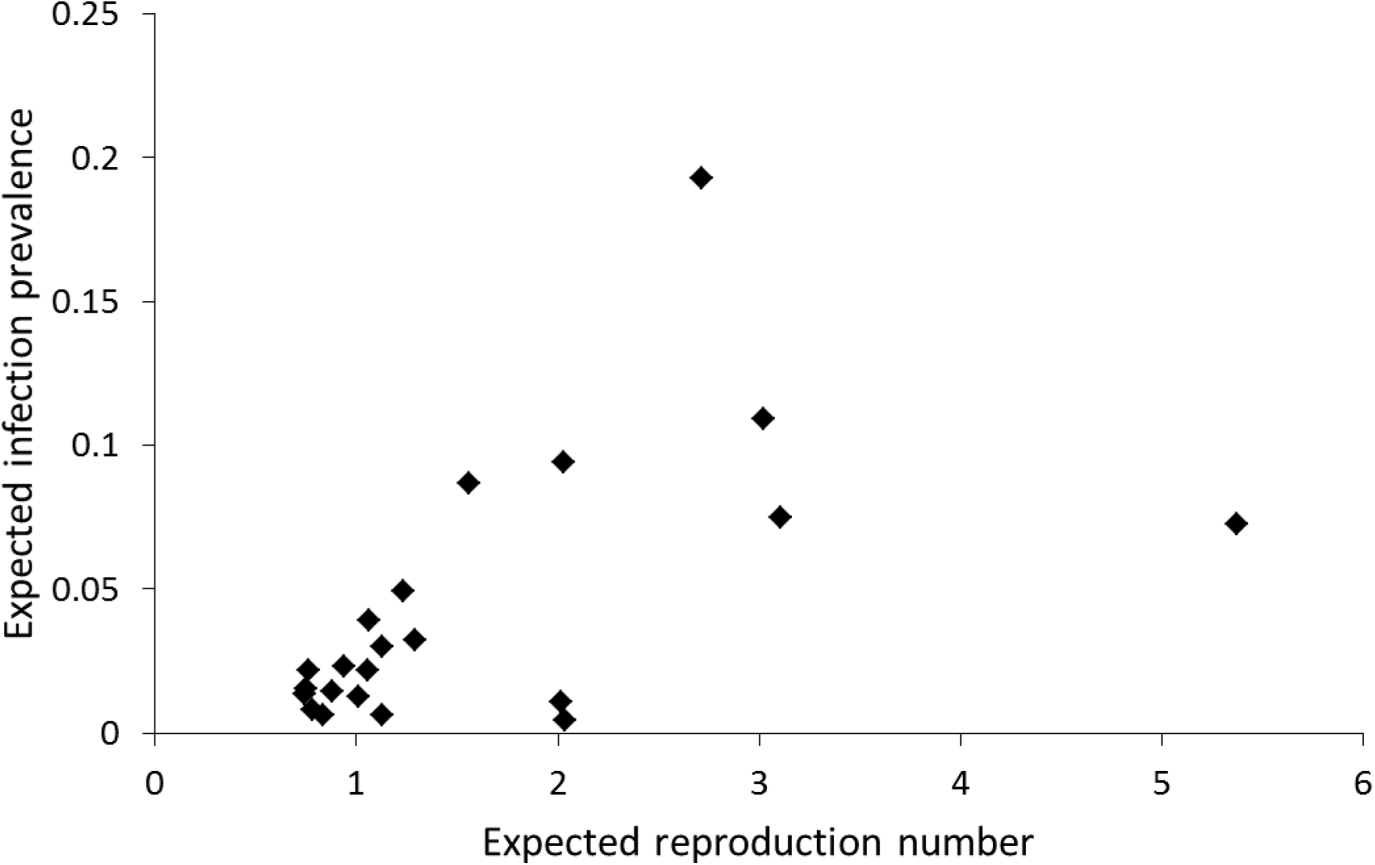
Threshold pattern. Expected within-flock infection prevalence against the “expected” within-flock reproduction number $\bar{\beta }\beta^{-1}R_{0}^{\text{w}}$. The expected within-flock infection prevalence is computed by weighing the genotype-specific detected infection prevalence in tested animals by the frequency of the genotype in genotyped animals. As this computation uses detected prevalence it assumes *Se* = 1.

In Fig 5 we illustrate how this robustness arises by plotting the expected within-flock infection prevalence when *Se* = 1 against the “expected reproduction number” defined by $\bar{\rho_{0}}\rho_{0}{}^{-1}R_{0}^{\text{w}}$. First, the results in Fig 5 display a threshold pattern close to $\bar{\rho_{0}}\rho_{0}{}^{-1}R_{0}^{\text{w}}=1$, and this threshold is the main determinant of the estimated $\bar{\rho_{0}}$ value. Second, we note that incorporating limitations in test sensitivity (*Se*<1) only affects the vertical scale and thus leaves the threshold pattern (and threshold location) unchanged. As a result, changing the value of *Se* has also little influence on the estimated $\bar{\rho_{0}}$.

## Discussion

Based on genotyping and scrapie testing data from culled flocks we have developed a relatively simple transmission model for calculating the basic reproduction number $R_{0}^{\text{w}}$ for within-flock scrapie transmission in The Netherlands. The model expresses the basic reproduction number for within-flock scrapie as a weighted average of genotype-specific susceptibilities, multiplied by a single overall transmission parameter. The value of overall transmission scale parameter is allowed to vary between flocks to account for random between-flock variation in non-genetic determinants such as management practice. Indeed, our estimation for the overall transmission parameter yields a distribution that spans across a substantial range (of about one order of magnitude). Risk factor analyses in the literature have found evidence for certain aspects of flock management practice promoting scrape incidence in affected flocks, namely lambing in group pens [31], and spreading sheep compost on the land and disposing of the placenta in the compost [32]. In principle, the variation observed in the overall transmission parameter may in part be due to certain simplifying modelling assumptions made; in particular, due to the assumption of genotype-independent relative infectiousness. We further note that (unknown) age distribution differences between flocks could in reality produce between-flock differences in test sensitivity (as this sensitivity depends on age since infection, which is strongly correlated with age itself). However, given the robustness of the mean and variance of the distribution of estimated values for the overall transmission parameter, we expect that our results would be only weakly affected by any such between-flock differences.

On the basis of only the flock-level genotype profile (i.e. in absence of prevalence data) our model, due to the random between-flock variation in the overall transmission scale parameter shown in Fig 2, provides no single $R_{0}^{w}$ estimate. However, the model enables the construction of an $R_{0}^{w}$ value distribution for a large population of flocks (e.g. a national population of flocks) from a sufficiently large data set of genotype profiles. Such a distribution can serve as a building block for a population-level scrapie transmission model that incorporates both within- and between-flock transmission. We note that some of the variation in the transmission scale parameter will arise from stochastic variation in the transmission dynamics, which is expected to yield variation in observed prevalence even between flocks that had identical parameter values.

The model was parameterized to apply to the classical scrapie strain(s) dominating in the Netherlands, to which the animals with a VRQ allele are most susceptible. In a small subset of culled flocks however, the prevalence pattern indicated the presence of an ARQ-adapted scrapie strain. For such a strain, the susceptibility pattern across non-ARR alleles is different [33], leading to a different dependence of the overall susceptibility of a flock on the genotype profile. A similar model as the one developed here could be developed for countries/breeds dominated by ARQ-adapted scrapie strain if a set of relative susceptibility parameter values is available.

Our model neglected scrapie-related mortality, and assumed that animal replacement rate did not differ between genotypes. Also we used a genotype-independent parameter for the sensitivity of the rapid test, motivated by age-at-onset results by Gubbins [25] that indicate that the incubation period distributions for the three genotypes without ARR allele (ARQ/ARQ, ARQ/VRQ, and VRQ/VRQ) are very similar. Inaccuracies introduced by these approximations would cause additional widening of the estimated distribution of values for the transmission scale parameter *ρ*_0_.

## Supporting Information

S1 Dataset (XLSX)

S1 Text (DOCX)
