# Supplementary material for: Investigation of a Simple Model for Within-Flock Transmission of Scrapie
Source: PLoS One. 2015 Oct 1;10(10):e0139436. doi: 10.1371/journal.pone.0139436 (PMC4591288; doi:10.1371/journal.pone.0139436)
Supplement: S1 Text — (DOCX) [file pone.0139436.s002.docx]

**Supporting Information file “S1 Text”**

to “Investigation of a simple model for within-flock transmission of scrapie” by T.J. Hagenaars and J.J. Windig.

*Derivation of Eq. (1)*

In a flock in endemic equilibrium, we have:

$\frac{dI_{\gamma}}{dt}=\beta g_{\gamma}S_{\gamma}\sum_{\gamma'} h_{\gamma'}I_{\gamma'}-\mu I_{\gamma}=0$.

Using $S_{\gamma}=1-I_{\gamma}$we may derive: $g_{\gamma}=\frac{I_{\gamma}}{1-I_{\gamma}}\frac{\mu}{\beta}\frac{1}{\sum_{\gamma'} h_{\gamma'}I_{\gamma'}}$. Using that, by definition, the relative susceptibility of the reference genotype equals one, i.e. $g_{\gamma_{\text{R}}}\equiv1$, we find:

$g_{\gamma}=\frac{g_{\gamma}}{g_{\gamma_{\text{R}}}}=\frac{I_{\gamma}}{I_{\gamma_{\text{R}}}}\left(\frac{1-I_{\gamma_{\text{R}}}}{1-I_{\gamma}}\right).$ (A)

Applying this result to the mean infection prevalence across all 69 flocks yields Eq. (1).

*Derivation of Eq. (2)*

After including the index $i$ to refer to the flock in question, equation (A) above reads:

$$g_{\gamma}=\frac{I_{\gamma,i}}{I_{\gamma_{\text{R}},i}}\left(\frac{1-I_{\gamma_{\text{R}},i}}{1-I_{\gamma,i}}\right).$$

For a given set of relative susceptibilities $g_{\gamma}$ this equation can be solved for $I_{\gamma,i}$, presenting a model prediction for the expected endemically infected proportions of the different genotypes:

$$I_{\gamma,i}=\frac{g_{\gamma}I_{_{\gamma_{\text{R}},i}}}{\left(1-I_{_{\gamma_{\text{R}},i}}\right)+g_{\gamma}I_{\gamma_{\text{R}},i}}$$

The expected proportions infected can be interpreted as probabilities $p_{\gamma,i}$, i.e. we may rewrite:

$p_{\gamma,i}=\frac{g_{\gamma}p_{\text{ARQ*/VRQ,}i}}{\left(1-p_{\text{ARQ*/VRQ,}i}\right)+g_{\gamma}p_{\text{ARQ*/VRQ,}i}}$.

*Derivation of Eq. (3)*

In endemic equilibrium we have:

$\frac{dS_{\gamma}}{dt}=\mu f_{\gamma}-\beta g_{\gamma}S_{\gamma}\sum_{\gamma'} h_{\gamma'}I_{\gamma'}-\mu S_{\gamma}=0$ (B)

Using that *h_γ_*=0 for the genotypes with at least one ARR allele and *h_γ_*=1 for the genotypes without ARR allele, and that by definition $i^{*}=\sum_{\gamma\in\Gamma} I_{\gamma}$, we may rewrite:

$$\sum_{\gamma} h_{\gamma}I_{\gamma}=\sum_{\gamma\in\Gamma} I_{\gamma}=i^{*}$$

Substituting $\sum_{\gamma'} h_{\gamma'}I_{\gamma'}$by $i^{*}$ in Eq. (B) and dividing by $\mu$ leads to:

$f_{\gamma}-\rho_{0}g_{\gamma}S_{\gamma}i^{*}-S_{\gamma}=0$.

We may thus solve:

$S_{\gamma}=\frac{f_{\gamma}}{\left(1+\rho_{0}g_{\gamma}i^{*}\right)}$. After substituting this result into the relationship $i^{*}=\sum_{\gamma\in\Gamma} I_{\gamma}=\sum_{\gamma\in\Gamma} f_{\gamma}-S_{\gamma}$we obtain Eq. (3).

*Approximate back-calculation of the genotype distribution*

For flocks with a recent history of selective breeding using only homozygote ARR rams, as evidenced by a negative FIS value, we seek to back-calculate, from the current genotype distribution, the “original” genotype distribution at times before the start of selective breeding. In the population we can distinguish between animals that were born before and animals born after the start of selective breeding strategy. We refer to the latter animals as “new-borns”. Assuming that the recent period of selective breeding is not much longer than two years, the genotype distribution $f^{n-b}$ amongst the new-borns is to a good approximation given by:

$$\begin{matrix} f_{R/R}^{n-b}=\overline{f}_{ARR}, \\ f_{R/S}^{n-b}=1-\overline{f}_{ARR}, \\ f_{S/S}^{n-b}=0. \end{matrix}$$

with $\overline{f}_{ARR}$ being the original ARR allele frequency. I.e. because of the selective breeding all animals inherit a R allele from the father and a proportion $\overline{f}_{ARR}$ inherits a R allele from the mother as well. (Note that in the first year of selective breeding, the genotype distribution of the new-borns is exactly given by the above equations. After two years, part of the new-borns will be added to the population of breeding ewes, thus changing the breeding ewes’ ARR frequency from the original one $\overline{f}_{ARR}$, turning the equations into an approximation. This approximation is thus worsening when the length of the period of selective breeding is increasing beyond two years.) If we denote by $\alpha$ the proportion of animals replaced by new-borns since the start of the selective breeding strategy, the genotype distribution $f^{c}$ at the moment of culling may be described in terms of $\overline{f}_{ARR}$. A proportion of 1-α of the animals at the time of culling thus consists of animals born before the selective breeding. For these we assume Hardy-Weinberg equilibrium, and consequently a proportion of ($\overline{f}_{ARR}$)² of these animals had the R/R genotype. Consequently the frequency of R/R at the time of culling can be described as follows:

$$f_{R/R}^{c}=\left(1-\alpha\right)\left(\overline{f}_{ARR}\right)^{2}+\alpha f_{R/R}^{n-b}=\left(1-\alpha\right)\left(\overline{f}_{ARR}\right)^{2}+\alpha\overline{f}_{ARR},$$

and likewise:

$\begin{matrix} f_{S/S}^{c}=\left(1-\alpha\right)\left(1-\overline{f}_{ARR}\right)^{2}+\alpha f_{S/S}^{n-b}=\left(1-\alpha\right)\left(1-\overline{f}_{ARR}\right)^{2}, \\ f_{R/S}^{c}=1-f_{R/R}^{c}-f_{S/S}^{c}. \end{matrix}$ (C)

After elimination of $\alpha$ from the first two equations in (C) we obtain the following cubic equation for $\overline{f}_{ARR}$:

The biologically relevant solution is given by

$$\overline{f}_{ARR}=\frac{1}{2}\left(1-f_{S/S}^{c}+f_{R/R}^{c}-\sqrt{\left(1-f_{S/S}^{c}+f_{R/R}^{c}\right)^{2}-4f_{R/R}^{c}}\right).$$

i.e. Eq. (4) in the main text.
